# The Efficiency of Allotransplant in a Case of Acute Biphenotypic Myeloid and B-Lymphoid Leukemia (MPAL Myelo/B NOS) That Presented Concurrently with a Mediastinal Granulocytic Sarcoma Co-Expressing Lymphoid Markers Complicated by Cardiac Tamponade

**DOI:** 10.3390/diagnostics16060953

**Published:** 2026-03-23

**Authors:** Alina Camelia Catana, Erzebeth Lazar Benedek, Ioan Zaharie, Liliana Mocanu, Geanina Mera, Cristina Popa, Lidia-Maria Mondoc

**Affiliations:** 1 County Clinical Emergency Hospital Sibiu, Bulevardul Corneliu Coposu 2-4, 550245 Sibiu, Romania; 2 Faculty of Medicine Sibiu, Lucian Blaga University of Sibiu, County Clinical Emergency Hospital Sibiu, Str. Lucian Blaga Nr. 2A, 550169 Sibiu, Romania; 3 County Clinical Emergency Hospital, 50 Gheorghe Marinescu St., 540136 Targu Mures, Romania

**Keywords:** biphenotypic acute leukemia, MPAL, mediastinal myeloid sarcoma, allotransplant, IVF

## Abstract

**Background and Clinical Significance**: Mixed-phenotype acute leukemia (MPAL) is a rare hematologic malignancy characterized by the co-expression of myeloid and lymphoid markers and is associated with poor prognosis. Myeloid sarcoma (MS), particularly in the mediastinum, is an uncommon extramedullary manifestation and is rarely reported in association with MPAL. **Case Presentation**: We report a rare case of mediastinal MS with biphenotypic features and pericardial extension occurring concurrently with MPAL, highlighting diagnostic challenges, therapeutic strategies, and long-term outcomes. We describe the clinical course, diagnostic workup, treatment, and follow-up of a 21-year-old woman who presented with cardiac tamponade secondary to a mediastinal mass. Histopathology and immunophenotyping established the diagnosis of mediastinal MS associated with MPAL (B/myeloid, NOS). Management included surgical cytoreduction, intensive induction chemotherapy, and consolidation with allogeneic hematopoietic stem cell transplantation (allo-HSCT) from an unrelated donor. Fertility preservation with oocyte retrieval, in vitro fertilization (IVF), and embryo cryopreservation was performed prior to conditioning. A focused literature review of MPAL cases with extramedullary involvement was conducted. The patient achieved complete remission following induction therapy and underwent allo-HSCT. Despite the historically poor prognosis of mediastinal MS and MPAL, she remains in sustained complete remission 13 years after diagnosis. A literature review identified only eight reported cases of MPAL with extramedullary disease, with mediastinal involvement described in a single case and allo-HSCT performed in only two patients. **Conclusions**: This case illustrates a rare presentation of MPAL with mediastinal myeloid sarcoma and cardiac tamponade, demonstrating that aggressive multimodal therapy including allo-HSCT may achieve durable remission even in high-risk presentations. Early multidisciplinary management and consideration of fertility preservation are essential in young patients.

## 1. Introduction

Mixed-phenotype (biphenotypic) acute leukemia (BAL/MPAL) is characterized by a dual-lineage manifestation, incorporating features of both myeloid and lymphoid malignancies. While its precise incidence remains elusive, it is estimated to account for approximately 5% of all acute leukemia diagnoses. The disease may manifest de novo or occur as a therapy-related malignancy (secondary MPAL) following exposure to cytotoxic agents or ionizing radiation administered for prior hematologic or solid organ neoplasms.

Although BAL may present across the entire age spectrum, a higher prevalence is noted in the adult population. Its clinical symptomatology is primarily dictated by the severity of bone marrow failure and resultant cytopenias. Morphologically, the blast population exhibits a spectrum of features bridging the transition between myeloblasts and lymphoblasts, rendering definitive morphological differentiation an arduous diagnostic challenge. Consequently, diagnosis relies heavily on flow cytometry; the blasts intensely express lineage-specific markers for both myeloid and lymphoid series.

Immunophenotypically, this entity is defined by the synchronous co-expression of myeloid and lymphoid antigens. The EGIL scoring system remains a cornerstone for BAL diagnosis, providing a robust framework for differentiating it from acute leukemias that merely exhibit aberrant lineage expression. Within the spectrum of BAL, the most prevalent immunophenotype is the B-lymphoid/myeloid lineage (60–70%), followed by T-lymphoid/myeloid (30%). Rarer configurations include B/T-lymphoid (4%) and trilineage manifestations (2%). Consistent with the evolving taxonomy of hematologic malignancies, these entities have been integrated into the WHO classifications (2008, 2016, and 2022) under the umbrella of ALAL. MPAL is an exceptionally rare clinical occurrence, with a documented incidence of approximately 0.35 cases per 1,000,000 person-years, representing only 0.6% to 2.4% of all acute leukemia cases [1].

The therapeutic management of MPAL remains exceptionally challenging, as a significant proportion of cases exhibit primary refractory disease during the induction phase. Even in instances where CR is attained, the clinical course is frequently marred by a high propensity for relapse. There is currently no standardized treatment algorithm; therapeutic selection is highly individualized, dictated by the blast morphology, immunophenotypic profile, and underlying cytogenetic or molecular aberrations. For patients who achieve CR, allo-HSCT is considered the definitive consolidative therapy. The overall prognosis remains suboptimal, with longitudinal studies estimating a 4-year survival rate of approximately 8%—a figure reflecting the inherent aggressiveness of the disease and the scarcity of large-scale clinical trials. Adverse prognostic indicators include age > 60 years, the presence of the Philadelphia chromosome (Ph+; BCR-ABL1 rearrangement), and failure to achieve CR following the initial induction cycle. Furthermore, the high expression of P-glycoprotein (P-gp), a key mediator of the multidrug resistance (MDR) phenotype, serves as a critical molecular marker for poor clinical outcomes [2].

Myeloid sarcoma (MS) is an extramedullary solid tumor composed of immature myeloid progenitors, occurring in approximately 2% to 8% of patients diagnosed with AML [1]. Its clinical presentation is heterogeneous; MS may manifest de novo, occur concurrently with systemic AML, or serve as the primary indicator of relapse in previously treated patients. Notably, it may also emerge as the initial clinical manifestation of blast transformation in patients with underlying myelodysplastic syndromes (MDSs) or myeloproliferative neoplasms (MPNs).

The topographic distribution of MS is extensive, frequently involving the bone, skin, lymph nodes, and soft tissues, as well as more sequestered sites such as the gastrointestinal tract, pleura, pericardium, pancreas, and testes [1,3,4].

Mediastinal myeloid sarcoma represents an exceedingly rare clinical entity that is frequently subject to diagnostic challenges, often being misidentified as primary mediastinal lymphoma. The association between MS—particularly when localized to the mediastinum—and MPAL is documented in only a limited number of cases within the literature. Historically, these cases are characterized by an aggressive clinical course, with reported average survival rates of approximately 19 months [5]. While MS is typically classified as granulocytic or monoblastic based on the predominant cell lineage, a biphenotypic immunophenotype in a mediastinal location remains an exceptional rarity.

Diagnosing primary (non-leukemic) myeloid sarcoma in the absence of peripheral blood or bone marrow involvement poses a formidable diagnostic challenge. Such cases may antedate acute leukemia by months or years and are frequently misidentified as primary mediastinal lymphomas. Non-leukemic MS is exceedingly rare, with an estimated incidence of 2 cases per 1,000,000 adults [1]. Data from the limited cohort of patients (aged 14 to 60 years) documented in the literature suggest an aggressive clinical course characterized by complex cytogenetic abnormalities and high early mortality rates, culminating in a dismal prognosis [6].

## 2. Case Presentation

**a.**  **Materials and Methods**

This study presents a detailed clinical case report of a 21-year-old female diagnosed with mixed-phenotype acute leukemia (MPAL), B/myeloid, not otherwise specified (NOS), associated with a mediastinal myeloid sarcoma co-expressing lymphoid markers and complicated by cardiac tamponade.

Diagnostic evaluation included clinical examination, a complete blood count, a peripheral blood smear, biochemical testing, a coagulation profile, and infectious disease screening. Imaging studies comprised transthoracic echocardiography (TTE), contrast-enhanced computed tomography (CT) of the chest, serial follow-up CT scans, positron emission tomography/computed tomography (PET/CT), and pelvic CT imaging. Bone marrow assessment included cytomorphology using May–Grünwald–Giemsa staining, cytochemical analysis (Periodic Acid–Schiff and myeloperoxidase reactions), multiparametric flow cytometry immunophenotyping, conventional cytogenetic karyotyping (20 metaphases analyzed), and molecular testing for BCR-ABL1 (major and minor transcripts), NPM1, and FLT3-ITD mutations.

Histopathological and immunohistochemical (IHC) analyses were performed on bone marrow biopsy and mediastinal tumor specimens using lineage-specific markers, including MPO, CD33, CD34, CD19, CD22, CD79a, CD3, CD5, CD10, CD20, CD43, and TdT. Diagnosis was established according to the 2016 and 2022 World Health Organization (WHO) classifications for acute leukemias of ambiguous lineage.

Therapeutic interventions included surgical cytoreduction and subtotal pericardiectomy, AML-directed induction chemotherapy (“3 + 7” regimen), high-dose cytarabine consolidation, and allogeneic hematopoietic stem cell transplantation (allo-HSCT) from a 10/10 HLA-matched unrelated donor following treosulfan-based conditioning. Fertility preservation was performed prior to transplantation via oocyte retrieval, in vitro fertilization, and embryo cryopreservation.

Written informed consent was obtained from the patient for publication. Data are available from the corresponding author upon reasonable request, in accordance with institutional and national ethical standards. No generative artificial intelligence tools were used in the design, data analysis, or interpretation of this study.

**b.**  **Detailed Case description**

We present the case of a 21-year-old female patient with no known history of toxic exposure who was admitted in May 2013 to the Cardiology Department. The patient presented with dyspnea, orthopnea, and anterior chest pressure; symptoms had an insidious onset one month prior and acutely worsened 48 h before admission. Upon clinical examination, the patient was afebrile and presented with Grade II obesity. Physical findings included jugular venous distension, polypnea (respiratory rate: 36 breaths/min), and tachypnea. Lung auscultation revealed diminished breath sounds with prolonged expiration. Cardiovascular assessment showed tachycardia (heart rate: 130 bpm), blood pressure of 120/70 mmHg, and muffled heart sounds. No hepatosplenomegaly was noted. The peripheral oxygen saturation (SaO_2_) was 89% on room air. Arterial blood gas (ABG) analysis confirmed hypoxemia (low pO_2_) and hypercapnia (increased pCO_2_).

The complete blood count (CBC) revealed a white blood cell (WBC) count of 5340/mm^3^, hemoglobin (Hb) of 12 g/dL, and a platelet count of 330,000/mm^3^. The WBC differential was 88% neutrophils, 0% eosinophils, 0% basophils, 8% lymphocytes, and 4% monocytes. Peripheral blood smear analysis showed no atypical cells. The inflammatory markers and coagulation profile were as follows: an erythrocyte sedimentation rate (ESR) of 22 mm/h, fibrinogen (Fbg) of 227 mg/dL, APTT of 27.3 s, PT of 12.4 s, INR of 1.15, and negative D-dimers. Tumor marker screening (TMF) was negative.

Biochemical analysis demonstrated elevated alanine aminotransferase (ALT) at 81 U/L (normal range: 3–43 U/L), while aspartate aminotransferase (AST) was 31 U/L. Alkaline phosphatase (ALP) and gamma-glutamyl transferase (GGT) were within normal limits. Renal function tests, uric acid levels, and serum protein electrophoresis were unremarkable. Serum iron was measured at 46.6 µg/dL.

Transthoracic echocardiography (TTE) revealed that the left ventricular ejection fraction (LVEF) was preserved at 63%. Morphological findings included a slightly thickened interventricular septum (IVS) of 12 mm (normal range: 7–11 mm) and a posterior wall thickness (PWT) of 11 mm. Wall motion was globally preserved. A large, circumferential pericardial effusion was identified, measuring 30 mm anterior to the right ventricle (RV). No evidence of right-sided chamber collapse (diastolic collapse) was observed, suggesting the absence of acute cardiac tamponade at the time of examination.

**Figure 1:**
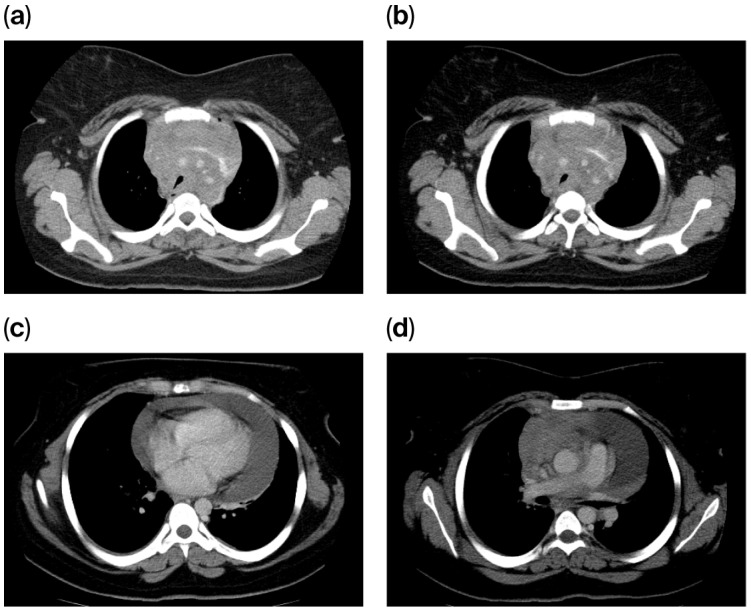
(**a**–**d**) CT scan at initial diagnosis (May 2013), demonstrating a mediastinal mass and a significant pericardial effusion measuring 3.7 cm in maximum depth.

The chest computed tomography (CT) scan performed on 15 May 2013, demonstrated extensive mediastinal lymphadenopathy involving all nodal stations, resulting in the encasement and compression of the mediastinal vascular structures. A moderate-to-large circumferential pericardial effusion was noted, with a maximum thickness of 3.7 cm; however, no pleural effusions were identified. The pulmonary parenchyma was clear, with no evidence of focal or consolidative lesions. Visualized subdiaphragmatic structures, including the liver, spleen, and pancreas, appeared unremarkable, and no abdominal lymphadenopathy was detected (Figure 1).

The immediate therapeutic interventions considered were either tumor debulking combined with pericardiectomy or a transthoracic biopsy of the mediastinal mass alongside pericardial drainage. Given the patient’s critical status in acute cardiorespiratory failure, surgical management was prioritized. Due to the voluminous pericardial effusion with impending cardiac tamponade and the extensive infiltration of adjacent vasculature by the mediastinal tumor, a partial tumor resection and subtotal anterior pericardiectomy were performed. Based on the initial clinical presentation and preliminary histopathological evaluation, a tentative diagnosis of T-cell lymphoblastic lymphoma (T-LBL) was proposed.

The postoperative course was favorable, with peripheral oxygen saturation (SaO_2_) normalizing to 100%, although sinus tachycardia persisted at 120 bpm; no further signs of cardiorespiratory failure were noted. Cytological analysis of the pericardial fluid smears revealed low cellularity, consisting of small-to-medium-sized lymphoid elements. Some cells exhibited prominent nucleoli—suggestive of lymphoblasts—while others showed condensed nuclei. These findings were consistent with a diagnosis of lymphomatous pericarditis.

From 26 May to 21 June 2013, the patient was readmitted to the Hematology Department for comprehensive diagnostic evaluation and staging, pending the final immunohistochemistry (IHC) results.

Laboratory results showed that the repeat complete blood count and biochemical profiles remained stable compared to prior evaluations. A comprehensive infectious disease screening was performed, revealing negative results for HBsAg, HIV, HTLV, and Treponema pallidum antibodies, while HBsAb was positive, indicating prior immunity. Serological testing for Toxoplasma, Cytomegalovirus, and Epstein–Barr Virus showed an IgG-positive and IgM-negative profile in all cases, consistent with latent immunity without evidence of acute infection.

The bone marrow aspirate demonstrated a hypercellular marrow characterized by 43% blast cells. These blasts were medium-to-large and exhibited a high nucleocytoplasmic ratio, frequently presenting lobulated or indented nuclei with one to three visible nucleoli and weakly basophilic, agranular cytoplasm. The granulocytic series was quantitatively reduced to approximately 25%, consisting primarily of myelocytes and mature granulocytes, while the megakaryocytic series remained normoplastic with evidence of active thrombopoiesis.

Cytochemical analysis demonstrated that 38% of the blast population was PAS-positive, exhibiting a fine granular pattern with a calculated glycogen score of 0.8. The myeloperoxidase reaction, performed using ortho-toluidine, identified peroxidase positivity in 22% of the blasts.

**Figure 2:**
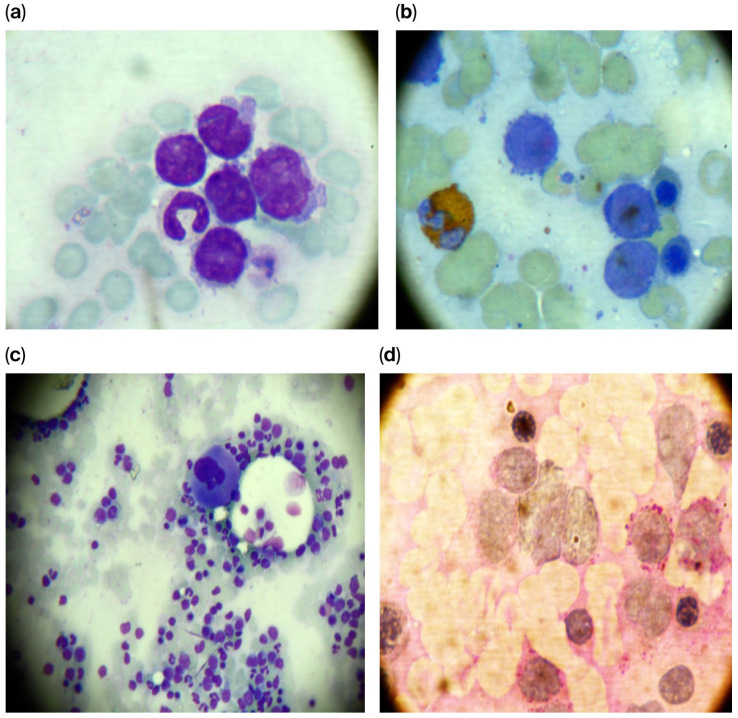
(**a**–**d**) Cytomorphological and cytochemical assessment of the bone marrow aspirate using May–Grünwald–Giemsa (MGG), Periodic Acid–Schiff (PAS), and myeloperoxidase (MPO) stains. (**a**) Bone marrow aspirate (MGG stain, ×1000 magnification): a cluster of five blasts exhibiting immature chromatin and prominent nucleoli. (**b**) Bone marrow aspirate (MPO stain, ×1000): a group of three blasts, two of which exhibit strong myeloperoxidase positivity. (**c**) Bone marrow aspirate (MGG stain, ×200): hypercellular marrow showing a dense infiltration of pleomorphic blasts exhibiting significant anisocytosis. (**d**) Bone marrow aspirate (PAS stain, ×1000): a heterogeneous blast population demonstrating both PAS-positive and PAS-negative reactions.

Based on these findings, the bone marrow presented features consistent with acute leukemia. Given the cytochemical profile, the differential diagnosis included MPAL Myeloid/B-cell, NOS according to the WHO 2008 classification. Alternatively, considering the presence of the voluminous mediastinal mass, these findings could suggest bone marrow involvement in the context of a myeloid sarcoma with associated biphenotypic myeloid–lymphoid acute leukemia (Figure 2).

Bone marrow immunophenotyping using CD45/SSC gating identified a blast population of 54% with a distinct immature phenotype. The blasts demonstrated strong positivity for CD33 (90%) and CD38 (100%), while CD34 was expressed in 55% of the population. Intracellular MPO was positive in 26% of the blasts. Markers of the lymphoid lineage showed partial or weak expression, with CD19 positive in 25% and surface CD22 (sCD22) in 15% of the cells, while HLA-DR showed variable expression in 30% of the population. The blasts were negative for monocytic markers (CD14, CD64), megakaryocytic markers (CD41, CD61), and erythroid markers (CD235). Furthermore, the population was negative for CD10, CD20, CD5, CD4, CD8, intracellular CD3 (icCD3), and surface light chains (Kappa and Lambda).

**Figure 3:**
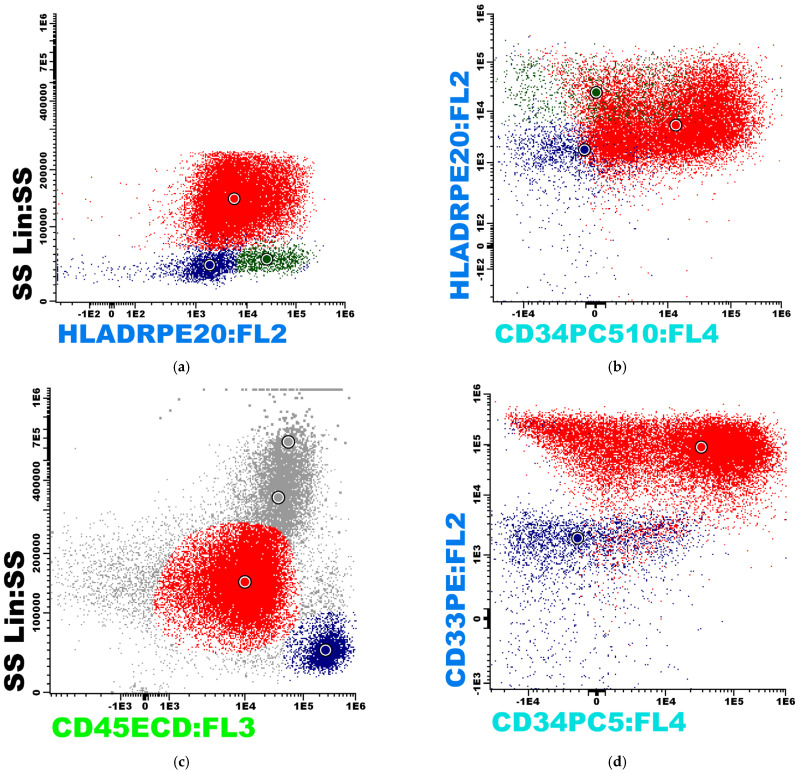

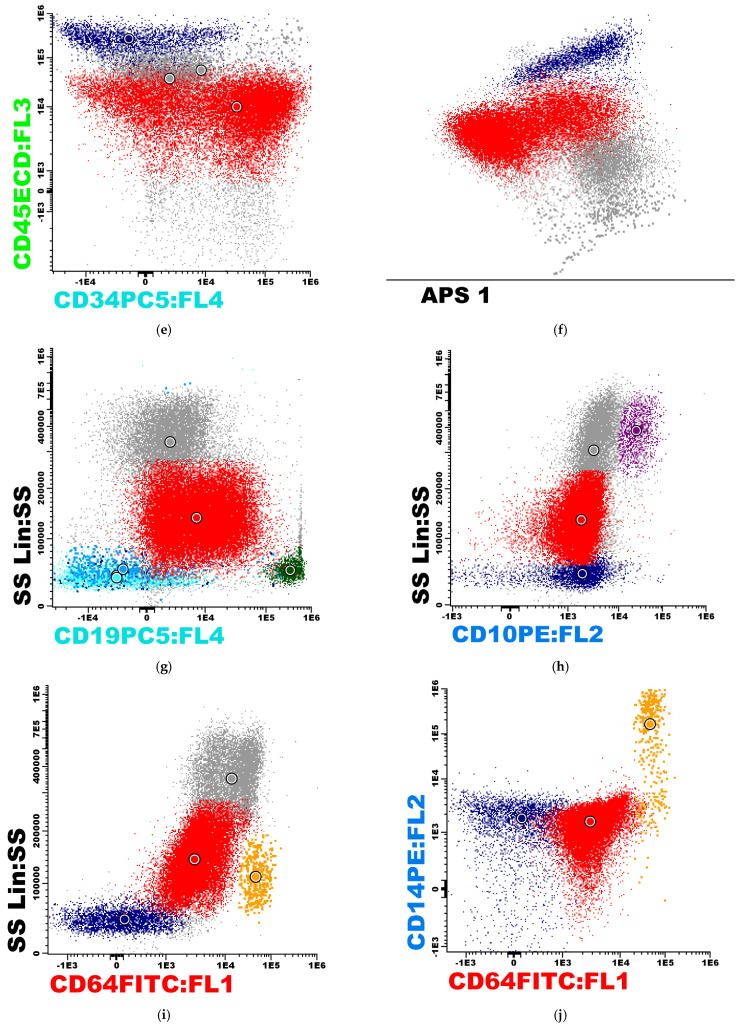
(**a**–**j**). Bone marrow aspirate and biopsy findings demonstrating MPAL, B-cell/myeloid, and NOS.

The final diagnosis was established as MPAL with myeloid and B-lymphoid lineages. This was supported by an EGIL score of 3 points for the myeloid lineage, derived from MPO positivity (2 points) and CD33 expression (1 point), alongside 3 points for the B-lymphoid lineage, based on CD22 (2 points) and CD19 (1 point) expression. According to both the 2016 and 2022 WHO classifications, the case is categorized as MPAL myeloid/B-lymphoid, NOS. The use of an additional immunophenotyping panel is recommended, incorporating markers such as CD117, CD13, CD11b, CD11c, CD16, CD36, CD56, CD2, CD7, and CD79a. Although the EGIL scoring system for acute leukemias is established using bone marrow aspirate or peripheral blood samples via flow cytometry (Figure 3), theoretically, this score could also be applied to a mediastinal sarcoma when it presents as a medullary component of a mixed-phenotype acute leukemia (MPAL); however, its interpretation in such cases is considerably more difficult. Consequently, we restricted the application of the EGIL score strictly to the bone marrow examination.

Histopathological and immunohistochemical evaluation of the bone marrow revealed a biopsy fragment with partially preserved architecture and marked hypercellularity, estimated at 70–75%. The cellular distribution was uneven, showing sparse normocellular regions alongside areas of diffuse infiltration by a blast population. Most of these blasts exhibited myeloid features and a tendency toward maturation, primarily arrested at the metamyelocyte stage with diminished terminal maturation. Lymphoid precursor elements were also identified within the infiltrate. The erythroid lineage appeared disorganized, poorly differentiated, and significantly underrepresented, with precursors showing impaired maturation. Megakaryocytes were rare, with some demonstrating dysplastic features. Additionally, focal reticulin fibrosis was observed, characterized by both fine and thickened fibers, while iron deposits were absent.

IHC analysis demonstrated that CD3 and CD5 were expressed in a subset of the lymphoid elements, while CD10 was positive in the majority of this population. CD20-positive B lymphocytes were also identified, though they were less frequent than the CD3-positive T-cell component. Additionally, the lymphoid cells were negative for CD23 but showed positivity for Bcl2.

**Figure 4:**
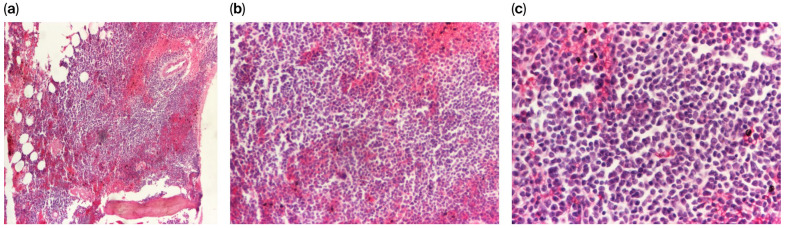
(**a**–**c**) Histopathological assessment of the bone marrow biopsy at varying magnifications (40×, 100×, and 200×) using hematoxylin and eosin (H&E) staining.

**Figure 5:**
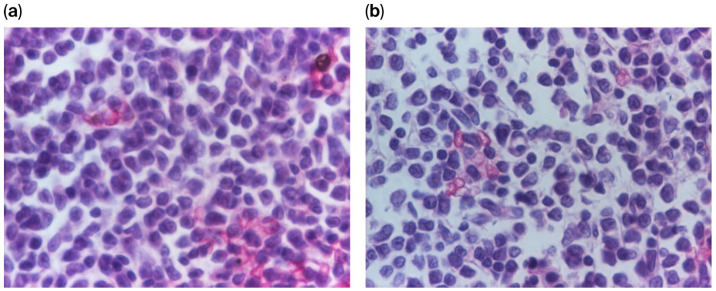
(**a**,**b**) High-magnification (400×) photomicrographs illustrating a dimorphic blast population, characterized by a heterogeneous mixture of lymphoblasts and myeloblasts.

The histopathological and immunohistochemical findings are consistent with a diagnosis of biphenotypic acute leukemia (Figure 4 and Figure 5).

**Figure 6:**
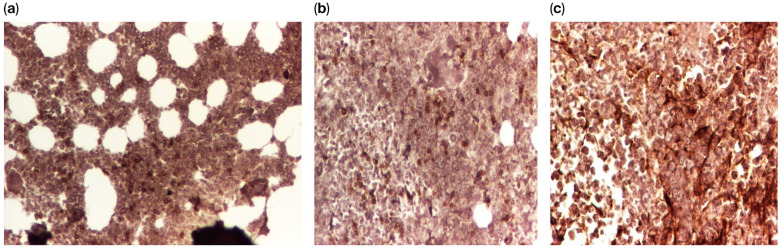
(**a**–**c**) IHC analysis of the lymphoid population. The atypical lymphoid cells demonstrated negative expression for CD5, CD20, and CD23, which were positive only in the background of residual normal lymphocytes. CD10 showed focal and heterogenous positivity, while Bcl-2 exhibited diffuse expression. These findings exclude chronic lymphoproliferative disorders or lymphomas, raising suspicion of acute lymphoblastic leukemia. Additionally, the presence of numerous blasts with impaired maturation (arrested predominantly at the myelocyte stage, with infrequent metamyelocytes) and a lack of terminal differentiation suggest a concomitant myeloproliferative component. According to the WHO classification, the findings are consistent with MPAL, Myeloid/B-cell, and NOS. Scale bar: 100×.

Histopathological analysis of CD34 and CD43 was performed. The proliferation showed weak, focal positivity for CD79a, while remaining negative for the T-cell markers CD3 and CD5, the thymocyte marker CD1a, terminal deoxynucleotidyl transferase (TdT), and the monocytic marker CD68. These histopathological and immunohistochemical findings are compatible with a myeloid sarcoma exhibiting partial expression of lymphoid markers, specifically CD79a (Figure 6).

Cytogenetic analysis of 20 metaphases revealed a normal female karyotype (46, XX) with no structural or numerical abnormalities. In molecular biology testing, the patient was negative for the BCR-ABL major and minor transcripts, as well as for NPM1 and FLT3-ITD mutations.

In 2013, we did not have the possibility of performing NGS for our case. Theoretically, NGS can also be performed on paraffin-embedded tissue from the mediastinal biopsy or bone marrow trephine; however, the decalcification process used for the marrow and the potential DNA degradation in older paraffin blocks represent significant technical limitations. Consequently, retrospective sequencing of these tissues was not pursued, despite the fact that it would have represented a clinical challenge and could have provided valuable insights into the favorable prognostic factors underlying the patient’s survival.

Pulmonary imaging showed a significantly enlarged superior mediastinum with the left upper arc convex toward the lung field, consistent with persistent lymphadenopathy and signs of sternal osteosynthesis.

Follow-up echocardiography demonstrated preserved LVEF with an ejection fraction of 60%. Microbiological cultures from the sternal wound were positive for *Klebsiella* species and *Candida* species.

The final diagnosis was established in accordance with the 2008 WHO classification as MPAL myeloid/B, NOS. This was associated with a granulocytic mediastinal sarcoma exhibiting co-expression of lymphoid markers.

The clinical course was complicated by mediastinal compression syndrome, as well as acute respiratory and cardiac failure. The patient’s surgical history included a partial tumor reduction and a pericardiectomy performed for cardiac tamponade. Additionally, the postoperative period was marked by a localized infection of the sternal wound with *Klebsiella* and *Candida* species.

Several therapeutic dilemmas arose during the management of this case. One primary concern was whether the induction regimen should prioritize the protocols for AML or those for ALL. Furthermore, the optimal role and timing of consolidative strategies, such as radiotherapy and hematopoietic stem cell transplantation, remained to be established. Finally, it was debated whether surgical resection followed by chemotherapy and stem cell transplantation would be sufficient for the long-term control of the mediastinal tumor, or whether localized radiotherapy would be required as an additional consolidative measure.

The multidisciplinary team opted for a treatment strategy targeting AML to address both the mediastinal myeloid sarcoma and the myeloblastic cell population. This plan was to be followed by Allo-HSCT, with localized radiotherapy reserved as a subsequent consolidative measure should post-transplant PET/CT imaging indicate residual metabolic activity.

The patient received two cycles of “3 + 7” induction chemotherapy, followed by four consolidation cycles of high-dose cytarabine (HD-AraC): Cytarabine at 3 g/m^2^ every 12 h on days 1–3 (total dose 27 g) and Idarubicin at 12 mg/m^2^ on days 1–3 (total dose 60 mg). CR was achieved following the first induction cycle and was successfully maintained throughout the subsequent treatment. HLA typing revealed that the patient was haploidentical with one brother and completely mismatched with the other tested brother.

Following the third cycle in September 2013, a follow-up computed tomography scan demonstrated two residual superior mediastinal lymphadenopathies in the pre- and paratracheal regions, measuring 14 mm and 19 mm in diameter, respectively. No other pathological findings were identified. The final treatment cycle, the fifth, was completed on 5 December 2013.

In light of the hormonal assay results, oocytes were harvested and fertilized using sperm collected from the patient’s fiancé. After assessing the quality of the resulting embryos, they were cryopreserved for future implantation. Oocyte harvesting was performed after the completion of the chemotherapy cycles but prior to the hematopoietic stem cell transplantation. During the interval between oocyte retrieval and the Allo-HSCT, the patient received maintenance therapy consisting of oral Methotrexate and Purinetol. On 11 April 2014, an allo-HSCT was performed from a 10/10 HLA-matched unrelated donor (MUD), after conditioning with Treosulfan and Cyclophosphamide and ATG (anti-thymocyte globulin). Between 3 April and 6 May 2014, the patient was hospitalized in the Bone Marrow Transplant Unit.

Graft-versus-host disease (GVHD) prophylaxis consisted of Methotrexate administered on days +1, +3, and +6, followed by a regimen including Tacrolimus, Cyclosporine, and Methylprednisolone.

The patient developed mild cutaneous GVHD and low-grade hemolysis secondary to major ABO incompatibility (Recipient: A+; Donor: B+). During the aplastic phase, the patient developed diarrheal syndrome and acute bronchitis, necessitating broad-spectrum antimicrobial therapy (antibiotic, antiviral, and antifungal agents) and intravenous immunoglobulin support. On day +6, the clinical course was complicated by recurrent hematemesis and menometrorrhagia, which were managed with blood product transfusions and recombinant activated factor VII (rFVIIa; NovoSeven).

At the time of discharge, the complete blood count (CBC) was within normal limits, with a white blood cell (WBC) count of 5830/mm^3^, hemoglobin of 10 g/dL, and a platelet count of 125,000/mm^3^. Post-transplant immunosuppression consisted of Tacrolimus and methylprednisolone; Tacrolimus was subsequently discontinued six months following the allo-HSCT. Bone marrow assessment at the three-month follow-up confirmed complete donor chimerism.

The post-transplantation period was complicated by the development of iatrogenic Cushing syndrome, characterized by obesity and striae distensae across the thoracic and abdominal regions. Furthermore, the patient’s clinical course was marked by mild cutaneous GVHD and transient hepatic dysfunction. During the initial six months following transplantation, the patient also experienced recurrent polymicrobial infections, requiring ongoing clinical management.

Follow-up imaging studies, including chest CT scans performed in August 2014 and February 2015, demonstrated no evidence of mediastinal, hilar, or axillary lymphadenopathy.

In March 2015, a PET/CT scan identified a metabolically active left supraclavicular lymphadenopathy, consistent with an inflammatory etiology. The exam also noted post-sternotomy cerclage with slight, diffuse FDG uptake. Consequently, radiotherapy was deferred.

In May 2015, the patient presented with right inguinal pain; however, inguinal hernia was clinically ruled out. The presence of metallic sternal sutures precluded the magnetic resonance imaging (MRI) required to investigate a clinical suspicion of avascular necrosis (AVN) of the femoral head. The patient was considered at high risk for AVN due to multifactorial predispositions, including obesity, prolonged corticosteroid therapy, and a history of hematopoietic stem cell transplantation.

**Figure 7:**
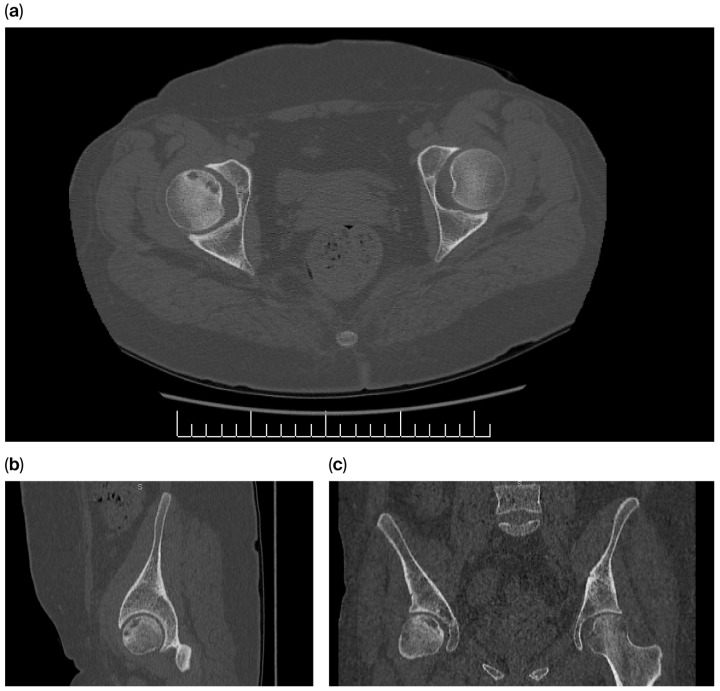
(**a–c**) Pelvic CT images demonstrating Stage III AVN of the right femoral head, characterized by subchondral radiolucency and cortical indentation.

Pelvic CT imaging revealed Stage III osteonecrotic changes in the right femoral head. The findings included areas of osteosclerosis and confluent lesions bordering a subchondral radiolucent cystic area. A focal breach in cortical continuity with a subsequent minor indentation was observed, consistent with Stage III AVN. No pathological changes were noted in the right acetabulum or the contralateral hip joint (Figure 7).

Surgical intervention was deferred. The patient initiated a conservative management regimen consisting of swimming and a controlled weight-loss program, achieving a total reduction of 20 kg. These interventions resulted in significant alleviation of the coxofemoral pain, which subsequently resolved. Follow-up indicated bone remodeling with preservation of the femoral head architecture, thereby precluding the need for total hip arthroplasty.

**Figure 8:**
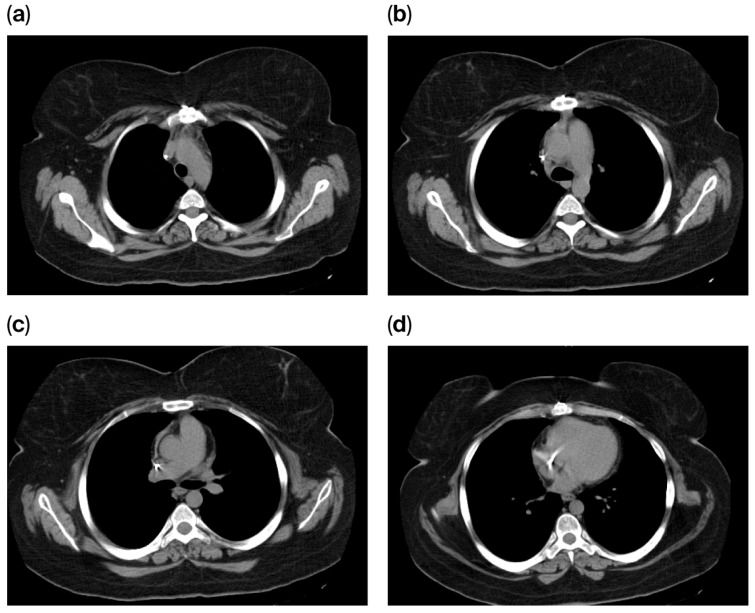
(**a–d**) Chest CT scan demonstrating a normal postoperative appearance.

The chest CT scan performed in May 2016 demonstrated sternal cerclage with no evidence of suspicious lesions within the pulmonary parenchyma. No suspicious mediastinal, pleural, or pulmonary abnormalities were identified, and the visualized portions of the liver, pancreas, and spleen appeared unremarkable. Furthermore, no suspicious lesions were noted within the visualized skeletal segments, leading to the conclusion of a normal postoperative CT appearance (Figure 8).

Despite the aggressive nature of this pathology, which is typically associated with low rates of CR, overall survival (OS), and progression-free survival (PFS) in the literature, the patient remains alive and in CR. Currently, she is 13 years post-diagnosis and 12 years post-allogeneic transplantation, with no evidence of mediastinal tumor recurrence. Regarding the patient’s long-term survival, we consider that this success resulted from a multifactorial synergy rather than solely the absence of high-risk molecular aberrations. The near-complete surgical resection of the mediastinal mass, which significantly reduced the tumor burden, followed promptly by aggressive chemotherapy—to which the patient achieved a complete and sustained response—represents a critical factor in this favorable outcome. Furthermore, the absence of significant complications, such as severe infections or hemorrhages, during induction and high-dose consolidation cycles allowed for the maintenance of optimal dose intensity. Finally, the early identification of an unrelated donor and seamless access to a bone marrow transplant center immediately following the completion of chemotherapy were additional key milestones in the successful management of this case.

Regarding her reproductive goals, the patient underwent two embryo transfer procedures using cryopreserved embryos five years after the transplant; unfortunately, these attempts did not result in pregnancy. Although we cannot state this with absolute certainty, the two unsuccessful embryo transfer procedures performed five years post-transplant were likely attributable to a compromised uterine environment. The intensive conditioning regimen, involving high-dose chemotherapy and hematopoietic stem cell transplantation, presumably led to impaired uterine vascularization and reduced endometrial receptivity. Furthermore, no primary embryonic abnormalities were identified as the cause of the implantation failures. While the option of surrogacy is being considered, it has been postponed for the time being.

## 3. Discussion

The European Hematology Association (EHA) categorizes the extramedullary manifestations of myeloid neoplasms into four distinct clinical scenarios:

1. Myeloid sarcoma presenting synchronously with AML;
2. Extramedullary relapse of previously treated AML;
3. Blast phase transformation arising from an underlying MPN or MDS;
4. Isolated myeloid sarcoma, defined by the absence of concurrent bone marrow or peripheral blood involvement [1,7].

Initially termed granulocytic sarcoma by Rappaport in 1966 [8], MS remains a rare and clinically aggressive manifestation of myeloid neoplasia, characterized by poor prognostic outcomes and limited survival rates. The clinical trajectory of MS is highly variable: it may present as an isolated (primary) entity in the absence of medullary involvement [9], occasionally antedating systemic acute leukemia by months or years. Alternatively, MS may manifest synchronously with acute leukemia, typically mirroring the medullary myeloid phenotype (e.g., monoblastic differentiation).

In cases associated with mixed-phenotype (biphenotypic) leukemia, the immunohistochemical profile of the sarcoma may either demonstrate a phenotypic identity with the bone marrow or, more rarely, represent only a single lineage—a phenomenon known as lineage discordance. Furthermore, MS may emerge as a sequela to acute leukemia during clinical remission or following allo-HSTC [10], in which instances it frequently serves as a harbinger of systemic leukemic relapse.

Granulocytic sarcoma occurs in an estimated 0.8% to 1% of all acute leukemia cases, with an even lower incidence reported in the context of MPAL [4,9]. Demographic data indicate that fewer than 10% of AML cases manifest in individuals under the age of 35. Furthermore, EMD—documented in approximately 1% of the AML population—demonstrates a predilection for the skeletal system, lymph nodes, and soft tissues; its manifestation within the mediastinum is considered an exceptional clinical rarity [9].

In the post-transplant setting, the clinical significance of extramedullary involvement is amplified. Isolated extramedullary relapse following allo-HSCT accounts for up to 15% of all post-transplant relapses [4]. This pattern of recurrence poses significant diagnostic and therapeutic challenges, often preceding or occurring in the absence of systemic medullary involvement.

MS may also manifest during the clonal evolution of other myeloid malignancies, including MDS, chronic myelomonocytic leukemia (CMML), and chronic MPN. According to data from the European Society for Blood and Marrow Transplantation (EBMT), the incidence of MS in pediatric cohorts with myeloid malignancies is estimated at 0.45%, with a post-transplant latency period ranging from 4 to 56 months.

In adult populations, the incidence varies by primary diagnosis: approximately 0.65% in AML and 0.22% in both chronic myeloid leukemia (CML) and MDS [10]. The specific association between mediastinal myeloid sarcoma and MDS is exceedingly rare; a comprehensive literature review spanning four decades (1981–2021) identified only 70 documented cases across 73 peer-reviewed articles [11]. This highlights the diagnostic scarcity and the clinical significance of reporting such presentations, particularly when associated with complex lineages.

Due to the inherent rarity of MS, the available literature is primarily limited to isolated case reports and small-scale retrospective cohorts. Currently, the most comprehensive benchmark for survival outcomes is derived from a retrospective analysis conducted at the First Affiliated Hospital of Zhengzhou University. This study evaluated 118 patients with MS treated between January 2010 and July 2021, reporting a notably dismal median OS of only 4 months (range, 1–51 months).

Crucially, survival outcomes were significantly superior in the subset of 11 patients who underwent allo-HSCT, where the median OS increased to 19 months (range, 8–51 months). Within this cohort, the maximum recorded survival duration was 51 months (approximately 4.25 years). In stark contrast, our case—demonstrating a sustained remission of 13 years—represents a remarkable outlier, more than doubling the maximum survival period documented in one of the largest available clinical series.

It is noteworthy that, in the Zhengzhou University cohort, five of the six long-term survivors received post-transplant maintenance therapy with decitabine. The graft-versus-leukemia (GvL) effect is significantly attenuated in extramedullary tissues compared to the bone marrow microenvironment [4,10], likely due to a localized reduction in immune surveillance. Decitabine, a hypomethylating agent, may counteract this immune evasion by modulating the post-transplant immune response. Evidence suggests that decitabine upregulates the activity of Natural Killer cells and cytotoxic CD8+ T cells, thereby enhancing the recognition and elimination of residual leukemic cells by donor-derived lymphocytes. This epigenetic modulation of the immune system may be a critical factor in achieving durable remission in high-risk presentations involving extramedullary myeloid sarcoma. The primary clinical objective of post-transplant maintenance therapy with hypomethylating agents, such as Decitabine, is the mitigation of systemic and extramedullary leukemic relapse.

In the comprehensive cohort from the First Affiliated Hospital of Zhengzhou University, the anatomical distribution of myeloid sarcoma was remarkably heterogeneous, involving the lymph nodes, soft tissues, spinal canal, gastrointestinal tract, genital system, pleura, skin, nasopharynx, lung, bone, brain, breast, orbit, gingiva, and parotid glands. While concurrent medullary involvement was documented in approximately 50% of the cases, mediastinal myeloid sarcoma was identified in only 4 out of 118 patients, representing a marginal incidence of 3.38%. Only one case was identified as primary mediastinal myeloid sarcoma, two cases presented concurrently with systemic acute leukemia, and one case manifested as an isolated mediastinal relapse. These findings align with broader epidemiological data, which report a remarkably low incidence of mediastinal granulocytic sarcoma at approximately 0.03%. Furthermore, when considering age-adjusted metrics, the incidence is estimated at 0.9%, with a median age at diagnosis of 59 years [12].

This underscores the diagnostic rarity of mediastinal presentations within the broader landscape of extramedullary myeloid malignancies and further contextualizes the clinical significance of our patient’s presentation and subsequent long-term survival.

Morphologically, the cellular composition of MS is characterized by significant cytological heterogeneity. The neoplastic infiltrate may be predominantly composed of promyelocytes, neutrophils, or myelomonocytes, while in other instances, monoblastic, erythroblastic, or megakaryoblastic features may prevail. Furthermore, the degree of maturation is highly variable, ranging from poorly differentiated blast populations to those exhibiting evidence of progressive myeloid maturation [13].

The definitive diagnosis of MS is established through comprehensive IHC profiling [1,14]. Lineage assignment relies on the strategic application of specific markers: MPO and CD117 serve as primary indicators of myeloid differentiation, while CD13 and CD68 characterize granulocytic, monocytic, and macrophagic subpopulations. The monocytic lineage is further delineated by lysozyme expression. Immature progenitors are identified by the presence of CD34 and TdT, whereas CD43 remains a sensitive, albeit less specific, marker for myeloid and lymphoblastoid lineages. Beyond phenotypic identification, the molecular landscape of granulocytic sarcoma provides critical prognostic insights. The most prevalent genetic alterations documented in the literature include mutations in KIT (16.6%), TET2 (14.6%), and NRAS (14.6%). Identifying these mutations is increasingly essential for risk stratification and the potential implementation of targeted therapeutic agents.

A case of mediastinal involvement was documented in a 9-year-old female with NPM1-mutated AML [15], highlighting that such presentations can occur even in the context of typically favorable-risk molecular markers. Furthermore, instances of p53-positive mediastinal granulocytic sarcoma have been cited; these cases are of particular clinical concern as they are frequently followed by the rapid onset of systemic AML, reflecting the genomic instability and aggressive disease kinetics associated with TP53 aberrations.

Genomic characterization plays a pivotal role in delineating the pathogenesis of these malignancies. Acute leukemia harboring the PICALM-MLLT10 fusion gene (formerly known as CALM-AF10) is a distinct subset typically manifesting as a mixed T-cell and myeloid phenotype [16]. This specific genetic aberration is often associated with an aggressive clinical course and a predilection for lineage ambiguity. Furthermore, high-risk molecular profiles are documented in cases such as the p53-positive mediastinal granulocytic sarcoma reported in a 25-year-old male presenting concurrently with systemic AML [9]. These findings underscore the importance of integrating molecular cytogenetics into the diagnostic workup to identify aggressive clones that may require more intensive therapeutic stratification.

Historical and contemporary literature further illustrates the heterogenous landscape of mediastinal MS. Early reports, such as those from 1984, established an initial association between mediastinal MS and acute promyelocytic leukemia (APL) [2,17]. More recent studies have identified even rarer associations, including a case of mediastinal MS occurring synchronously with acute megakaryoblastic leukemia, characterized by a KRAS mutation and highly complex cytogenetic abnormalities [18]. In a significant contribution from 2024, Wing-Yan Au described a landmark case of a 31-year-old male presenting with a primitive mediastinal MPAL, B/myeloid, manifesting as an isolated mediastinal mass. This case was driven by a TCF3-ZNF384 fusion, a genetic signature more commonly associated with lymphoid lineages. Notably, the patient achieved a complete radiological resolution of the mediastinal mass following standard “3 + 7” induction chemotherapy [19]. These reports underscore the necessity of comprehensive molecular profiling—including the detection of rare fusion genes—to refine the diagnosis and therapeutic strategy for mediastinal masses with ambiguous lineage.

Literature further documents the clinical heterogeneity and aggressive potential of high-risk molecular subtypes in extramedullary presentations. Carol Wang (2018) [20] reported a case of a 69-year-old male presenting with primitive T/myeloid MPAL manifesting as gastrointestinal involvement and pulmonary mass-forming lesions. This case was characterized by the presence of the Philadelphia chromosome, t(9;22)(q34.1;q11.2), resulting in the BCR-ABL1 fusion, and was associated with a rapidly aggressive clinical course following diagnosis [20]. In contrast, other documented extramedullary manifestations include cases of acute myeloid leukemia with t(8;21)(q22;q22), presenting as granulocytic sarcomas in atypical anatomical locations such as the nasopharynx and the external auditory canal [21]. While t(8;21) is typically associated with a more favorable prognosis in systemic AML, its manifestation as isolated or synchronous myeloid sarcoma requires vigilant diagnostic and therapeutic monitoring to mitigate the risk of systemic progression.

In contrast to the molecularly driven cases documented in the literature, our patient exhibited a normal karyotype and an absence of defining molecular aberrations.

Historically, the first description of this entity dates back to 1811 and was provided by Burns et al., who coined the term “chloroma” to describe the characteristic green hue of the tumor, a macroscopic feature attributed to high concentrations of the enzyme MPO [8,22]. In modern nomenclature, this is recognized as MS.

The mediastinal variant of MS exhibits significant morphophenotypic diversity and may be classified as monocytic, myeloid, or mixed-phenotype based on the predominant cellular differentiation [1,23,24]. Clinically, these mediastinal masses may manifest as an isolated primary lesion or occur synchronously with systemic acute leukemia, further complicating the diagnostic and therapeutic landscape.

The scarcity of MPAL presenting without medullary involvement is further underscored by the findings of Jeffrey Means et al., who identified only 18 such cases in their analysis. This cohort was predominantly pediatric (*n* = 11), with immunophenotypic distributions including B/myeloid (*n* = 7), T/myeloid (*n* = 2), and B/T-lymphoid (*n* = 2). Notably, all pediatric patients achieved CR following standard ALL-directed induction therapy. In striking contrast, only a single adult case of B/myeloid MPAL without bone marrow infiltration was documented in this series. That patient remained in remission for 19 months post-transplantation [23].

The clinical documentation of primary mediastinal MS with MPAL remains exceptionally sparse. To date, only twelve such cases have been recorded in the global literature, with a mere three cases specifically identifying the B/myeloid, NOS phenotype [1,19,23]. The sentinel report of non-leukemic MPAL B/myeloid, NOS was provided in 2018 by Teruhito Takakuwa, where the tumor demonstrated a complex immunophenotypic signature positive for MPO, CD10, CD19, and CD79a [1].

A critical diagnostic pitfall in the management of primary mediastinal MS is its frequent misidentification as mediastinal lymphoma. Statistical analyses indicate that 46% of mediastinal myeloid sarcomas are misdiagnosed at initial presentation [14,25]. These tumors are most commonly mistaken for diffuse large B-cell lymphoma (DLBCL) or T-lymphoblastic lymphoma, often resulting in the administration of inappropriate, lymphoma-specific therapeutic regimens [14,23,26,27]. This high rate of diagnostic discordance underscores the imperative for early, comprehensive immunohistochemical and molecular profiling to prevent therapeutic delay.

Extramedullary MS is renowned for its diagnostic mimicry, often presenting with clinical emergencies that obscure the underlying hematological malignancy. For instance, the literature cites a dramatic pediatric case of abdominal MS associated with biphenotypic leukemia, where intestinal infiltration led to complete thrombosis of the mesenteric artery [28]. Such cases exemplify the profound diagnostic confusion that can occur when the initial presentation mimics an acute surgical abdomen or a primary vascular catastrophe. While the mediastinal location is rare, other “sanctuary sites” present similar challenges; primary intracranial or intraspinal involvement remains an exceptional occurrence, often necessitating a complex differential diagnosis involving primary central nervous system tumors or infectious processes.

Furthermore, atypical presentations of MPAL involving biphenotypic blasts localized exclusively to the lymph nodes, in the absence of medullary infiltration, have been documented. Martin-Guerrero et al. categorized these rare clinical entities as “nonleukemic” MPAL [26]. This classification highlights a unique subset of ambiguous lineage leukemias that present as isolated extramedullary masses, mirroring the behavior of myeloid sarcoma but maintaining a complex, multi-lineage immunophenotype. The recognition of this “nonleukemic” variant is crucial, as it emphasizes that MPAL can exist as a compartmentalized disease, requiring systemic therapeutic approaches even when the bone marrow remains uninvolved.

Khanna G et al. documented a rare instance of MS of the thyroid gland occurring concomitantly with MPAL (B/myeloid, NOS). Similar to primary mediastinal tumors, the clinical manifestation of such lesions is primarily dictated by their expansive growth within confined anatomical compartments. Symptoms typically vary according to the specific mediastinal localization, tumor dimensions, and degree of extrinsic compression exerted on vital structures, including the major vasculature (leading to superior vena cava syndrome or cardiac tamponade) and the tracheobronchial tree (resulting in acute airway compromise) [16].

Clinical presentations involving acute cardiac tamponade as the sentinel manifestation of MS are exceedingly rare [29]. While isolated reports document cases of MS presenting with direct pericardial infiltration [30] or even infiltration of the myocardium [31], these manifestations are typically accompanied by significant pleural effusions and clinical features of superior vena cava syndrome due to mediastinal compression. Although concomitant pleural involvement in granulocytic sarcoma has been extensively documented in the literature [2], it is a noteworthy clinical feature that our patient exhibited no pleural involvement. This isolated mediastinal localization, resulting in a localized mass effect sufficient to induce life-threatening tamponade without widespread serosal disease, further distinguishes this case as a unique presentation within the spectrum of extramedullary myeloid malignancies.

The prognostic impact of anatomical localization in MS has been extensively elucidated by Goyal et al. [6], in a large-scale analysis of 746 MS cases, representing a marginal 0.8% of the total AML population (total cases of AML = 94,185). The study reported a median OS of 12.8 months, with distinct survival outcomes stratified by organ system: favorable prognosis (OS > 30 months)—involvement of the reproductive and gastrointestinal tracts; intermediate prognosis (OS 15–30 months)—localizations involving the head and neck, integumentary/breast tissues, and the renal/retroperitoneal axis; and poor prognosis (OS < 15 months)—involvement of the central nervous system, connective tissues, lymphoid organs, and the cardiopulmonary/mediastinal axis. Notably, within the high-risk cardiopulmonary and mediastinal subgroup (*n* = 32), the median age at diagnosis was 50.5 years, with a significantly compromised median survival of only 11 months. These findings underscore the aggressive nature of mediastinal MS and further contextualize the exceptional clinical course of our patient, whose survival duration significantly exceeds these established demographic and prognostic benchmarks.

Therapeutic strategies are predicated upon the specific cellular morphophenotype; in cases exhibiting a mixed phenotype, treatment regimens are typically aligned with protocols for acute myeloblastic or lymphoblastic leukemia. While the literature often favors ALL-type protocols for MPAL, we opted for an AML-oriented regimen in this specific case. This decision was primarily informed by the granulocytic morphology of the myeloid sarcoma, even though the co-expression of lymphoid lineage markers.

Surgical ablation of the sarcomatous mass warrants clinical consideration, particularly given the prevalence of this malignancy in younger demographics. Furthermore, meticulous diagnostic differentiation is essential, as the literature highlights frequent overlap and potential misdiagnosis with lymphoma, thymoma [17], and germ cell tumors [14,22,29].

Radiotherapy has been utilized as a palliative or adjunctive intervention [1,14] to achieve cytoreduction in instances of severe mediastinal compression syndrome or following an inadequate response to primary systemic chemotherapy. Evidence suggests, however, that radiation therapy in the absence of adjunctive chemotherapy or hematopoietic stem cell transplantation yields suboptimal response rates and poor leukemia-free survival [24]. Conversely, the integration of high-dose chemotherapy with transplantation continues to demonstrate the most favorable long-term survival outcomes [10,22]

Representing only 1–4% of acute leukemias [2,23,27], MPAL remains a therapeutic challenge, with evidence restricted to small-scale observational studies. The clinical course is frequently aggressive, with poor long-term outcomes necessitating intensive induction therapy followed by consolidation with allotransplantation. Diagnostic classification distinguishes between bilineal presentations (multiple discrete cell populations) and biphenotypic forms (singular populations with multi-lineage marker co-expression) [32], both of which contribute to the underlying heterogeneity and chemoresistance observed in this pathology.

Formally recognized in the 2008 WHO classification, these malignancies are defined as “leukemias that show no clear evidence of differentiation along a single lineage”. This classification consolidated diverse diagnostic entities characterized by lineage ambiguity under a unified framework.

Under the 2016 WHO revised criteria, MPAL is categorized into distinct subtypes based on cytogenetic and immunophenotypic profiles: (1) AUL; (2) MPAL with t(9;22)(q34.1;q11.2) (BCR-ABL1); (3) MPAL with t(v;11q23.3), KMT2A rearranged; (4) B/myeloid, NOS); and (5) T/myeloid, NOS [26].

The 2022 WHO classification [33] further refined the taxonomy of acute leukemias of mixed or ambiguous lineage, prioritizing molecular drivers over immunophenotypic characteristics. The current framework categorizes these entities into ALAL with defining genetic abnormalities (specifically BCR-ABL1 fusion, KMT2A rearrangement, or other emerging genetic alterations); ALAL defined by immunophenotype (which includes B/myeloid and T/myeloid subtypes, rare lineage combinations, and those NOS); and AUL, reserved for cases lacking lineage-specific markers.

The contemporary WHO classifications (2008, 2016, 2022) were preceded by the 1995 scoring system established by the EGIL for biphenotypic acute leukemia—a term that has since been largely subsumed under the nomenclature of MPAL. Notably, the transition to WHO criteria introduced a more stringent diagnostic threshold, refining the requirements for lineage assignment compared to the earlier EGIL parameters [34].

Comparative data suggest a therapeutic advantage for ALL-type regimens over AML-type protocols in this patient population, with reported response rates of 85% and 41%, respectively. This disparity extends to survival outcomes, where OS reached 139 months in the ALL-treated group compared to 11 months in the AML cohort [23,35,36,37]. However, evidence remains heterogeneous, as other reports document superior efficacy with myeloid-oriented induction in specific cases [1].

Allogeneic hematopoietic cell transplantation in MPAL is associated with superior clinical outcomes compared to conventional chemotherapy alone [2,27,38,39]. Prior to the widespread implementation of transplant-based consolidation, long-term survival rates were estimated at only 15–35% [27]. In a landmark analysis of 519 MPAL patients in first CR, Munker et al. reported a 3-year relapse rate of 31.4% and non-relapse mortality of 22.1%, yielding a 3-year leukemia-free survival of 46.5% and an OS of 56.3%. Regarding post-transplant complications, the incidence of acute GVHD was 32.5% at six months, while chronic GVHD reached 37.5% at three years—figures consistent with Center for International Blood and Marrow Transplant Research data [27]. Notably, the patient in our case exhibited neither acute nor chronic GVHD manifestations during the follow-up period.

A 2019 cohort study identified only 12 cases of BAL or ALAL among 377 patients, according to EGIL or WHO criteria. Survival analysis at five years demonstrated an OS of 51.1% and an EFS (Event Free-Survival) of 51.9%. Within this cohort, the incidences of AUL and MPAL were 0.5% and 1.3%, respectively. A critical distinction lies in the diagnostic rigor: whereas the EGIL scoring system incorporates a broad panel of lineage-specific markers, the WHO classification relies on a streamlined set of primary antigens, namely CD19 for the B-lineage, cytoplasmic CD3 for the T-lineage, and MPO for the myeloid lineage. Notably, AUL is defined by the absence of these WHO-specified antigens. In adult populations, the B-lymphoblastic/myeloid phenotype predominates (70%), followed by the T-lymphoid/myeloid phenotype (23–33%), while trilineage (B/T/myeloid) and dual-lymphoid (B/T) subtypes remain exceedingly rare [34].

MPAL is associated with an inferior prognosis compared to other acute leukemia subtypes, a disparity likely attributable to the inherent chemoresistance of multipotent progenitor cells. This resistance is driven by several mechanisms, including a quiescent, slow-proliferative state and the capacity for phenotypic plasticity, which allows blast cells to undergo lineage switching under therapeutic pressure. Furthermore, the over-expression of P-glycoprotein in these malignant clones confers a multidrug-resistant phenotype, significantly compromising the efficacy of standard induction regimens [2].

Therapeutic selection is traditionally guided by morphological characterization and cytochemical staining profiles. In a 2019 study, Hyun Gyung Lee reported CR rates of 100% for ALL-directed therapy versus 16.7% for AML-directed regimens [34]. These findings align with earlier evidence from Rubnitz et al. (83% vs. 52%), Gerr et al. (81% vs. 41%), and Matutes et al., all suggesting a therapeutic advantage for lymphoid-oriented induction. However, clinical outcomes remain variable; for instance, a Korean pediatric cohort demonstrated a more modest CR rate of 52%, despite the predominant utilization of ALL-type protocols, highlighting the influence of cohort-specific heterogeneity.

In an analysis by Santhosh Kumar Devadas involving 114 patients diagnosed with MPAL, the cohort was stratified by treatment intensity: 74% were treated with pediatric-inspired protocols, while 26% received adult-type regimens. Following induction therapy, a CR was achieved in 43 patients (65%). However, induction-related mortality was significant, with a 15% death rate (*n* = 10) reported during the initial treatment phase [40].

The clinical utility of hematopoietic stem cell transplantation remains a subject of considerable debate. Although post-transplant survival exceeds 50%, the absence of such intervention is associated with high mortality rates secondary to disease relapse. Over recent years, the survival outcomes for both pediatric and adult populations have demonstrated progressive improvement. Data indicate that intensive chemotherapy followed by allo-HSCT in pediatric cohorts yields survival rates of 80%; similarly, adolescents exhibit a two-year survival rate of 75%, contrasting sharply with the 17% observed in adult and geriatric patients. Investigations by Killick et al. and Gerr et al. demonstrated that the five-year EFS in acute leukemia of ALAL is inferior to that of ALL, yet superior to that of AML. Furthermore, survival in MPAL is markedly enhanced when transplantation is pursued, particularly if performed during the first complete remission using myeloablative conditioning and total body irradiation. While certain studies suggest that these outcomes are independent of MRD status, other evidence highlights MRD as a critical prognostic determinant for survival [41].

In a comprehensive literature review conducted by Kamal Kant Sahu, spanning cases from 1977 to 2013, the occurrence of myeloid sarcoma with pleural involvement was found to be exceedingly rare. Specifically, only six cases of mediastinal myeloid sarcoma associated with pleural involvement were identified, five of which were concomitant with acute leukemia [3].

MPAL is characterized by a median OS of 19 months and a five-year survival rate of 37%, utilizing therapeutic regimens tailored for both ALL and AML, followed by consolidation with allo-HSCT [1].

A comprehensive literature review identified eight cases of MPAL presenting with extramedullary involvement of either mixed or purely myeloid phenotype (Table 1). The anatomic distribution of these lesions included the pleura (*n* = 1), the lateral pharyngeal wall (*n* = 1), the thyroid gland (*n* = 1), peripheral lymph nodes (*n* = 2), abdominal lymph nodes (*n* = 1), and a single reported case involving the mediastinum. Notably, the case of mediastinal myeloid sarcoma exhibited disease progression to biphenotypic MPAL (T/myeloid) harboring the PICALM-MLLT10 fusion gene following four cycles of chemotherapy. In contrast, only the pleural and thyroid involvements were associated with MPAL (B/myeloid, NOS). Regarding therapeutic interventions, allo-HSCT was performed in only two instances; three cases followed ALL-directed protocols, while the remainder received AML-type induction. Clinical outcomes were consistently poor, aligning with the dismal survival rates reported in isolated series of MPAL, mediastinal myeloid sarcoma, and cases where myeloid sarcoma complicates biphenotypic acute leukemia. We posit that the therapeutic strategy employed in our patient, centered on a multidisciplinary team management approach, was the critical factor in achieving a successful outcome.

## 4. Conclusions

We report a rare clinical case of a 21-year-old female diagnosed with cardiac tamponade secondary to a mediastinal myeloid sarcoma co-expressing lymphoid markers, associated with mixed-phenotype acute leukemia, B/myeloid, NOS subtype. Management necessitated surgical debulking, intensive chemotherapy, and subsequent allo-HSCT from an unrelated donor. Despite the characteristically poor prognosis associated with both mediastinal myeloid sarcoma and biphenotypic acute leukemia, the patient remains in long-term remission 13 years post-diagnosis. The clinical presentation was highly atypical for MPAL for several reasons: the young age of the patient (21 years versus a literature-reported median of 54–56 years) and the presence of extramedullary disease. While EMD occurs in approximately 1% of acute leukemia cases, it typically involves the bones, lymph nodes, or soft tissues; mediastinal involvement is exceedingly rare. This case underscores that a multidisciplinary therapeutic approach, integrating surgical intervention with aggressive chemo-immunotherapy and transplantation, can achieve durable survival even in high-risk MPAL phenotypes.

## Figures and Tables

**Table 1:** Clinical characteristics of MPAL with extramedullary involvement: literature review and case comparison.

Reference	Year	Age/Sex	Myeloid Sarcoma Anatomic Site	Relationship with AML	MPAL Subtype	Treatment	OS
[42]	1999	32/M	Diffuse bulky lymphadenopathy	Concomitant	T/Myeloid MPAL (CML-BC)	“7 + 3” regimen Cyclophosphamide Etoposide Interferon Cyclosporine A	6 months post-biopsy 17 months post-blast crisis
[10]	2009	11/F	Pleural involvement	Concomitant Isolated pleural relapse 1 year post-allo-HSCT	MPAL, B/myeloid, NOS	ALL-BFM 95 (MR) protocol (1 block)—relapse in bone marrow after one year ALL-BFM 95 (HR) protocol Allo-HSCT (matched sibling donor)—isolated left pleural relapse MRC-12 AML protocol	27 months Death due to Gram-negative sepsis
[43]	2013	31/M	Lateral pharyngeal wall	Relapse 4 years post-transplant	MPAL, NOS	Chemoradiotherapy followed by allo-HSCT—isolated pharyngeal wall relapse Five cycles of salvage chemotherapy (Fludarabine/Cytarabine) Radiotherapy (15 Gy in 5 fractions) to the pharynx	Alive in CR at last follow-up
[44]	2016	61/M	Cervical and axillary lymphadenopathy	Concomitant	MPAL (T/Myeloid) t(1;5)(q23;q33)	Not reported	Not reported
[45]	2016	58/F	Thyroid involvement	Concomitant	MPAL, B/myeloid, NOS	ALL-type chemotherapy Hoelzer protocol concurrent with IT MTX	Not reported
[16]	2018	25/M	Primary mediastinal myeloid sarcoma	Medullary relapse following 4 cycles of chemotherapy	MPAL (T/myeloid) *PICALM-MLLT10* fusion	4 cycles of chemotherapy—PR BM relapse	Not reported
[28]	2022	11/F	Abdominal involvement	Concomitant	MPAL, T/myeloid (monoblastic), NOS	AML-BFM 2012 (AIE induction)	3 weeks Intestinal perforation MSOF
[26]	2023	72/M	Bilateral cervical Axillary Mediastinal lymphadenopathy	Bone marrow progression within 4 months	MPAL, T/myeloid, NOS	Refractory to ALL-type chemotherapy Salvage therapy with Azacitidine–Venetoclax Achieved CR	8 months post-diagnosis PD
**OUR CASE**	**2026**	**21/F**	**Mediastinal myeloid sarcoma with lymphoid marker co-expression**	**Concomitant**	**MPAL,** **B/myeloid, NOS**	**“7 + 3” Induction** **4 cycles of HiDAC + IDA** **Allo-HSCT**	**Alive in CR** **13-year follow-up**

## Data Availability

The data that support the findings of this case report are available from the corresponding author upon reasonable request. Due to privacy or ethical restrictions, some data may not be made publicly available to protect patient confidentiality. Data sharing is in compliance with the institutional and national ethical guidelines.

## Abbreviations

MPAL	Mixed-Phenotype Acute Leukemia
BAL	Acute Biphenotypic Leukemia
EGIL	European Group for the Immunological Characterization of Leukemias
ALL	Acute Lymphoblastic Leukemia
AML	Acute Myeloid Leukemia
Allo-HSCT	Allogeneic Hematopoietic Stem Cell Transplantation
WHO	World Health Organization
ALAL	Acute Leukemias of Ambiguous Lineage
AUL	Acute Undifferentiated Leukemia
MS	Myeloid Sarcoma
NOS	Not Otherwise Specified
IVF	In Vitro Fertilization
CR	Complete Remission
EMD	Extramedullary Disease
P-gp	P-Glycoprotein
MDR	Multidrug Resistance
MDS	Myelodysplastic Syndrome
MPN	Myeloproliferative Neoplasm
ALT	Alanine Aminotransferase
AST	Aspartate Aminotransferase
GGT	Gamma-Glutamyl Transferase
TTE	Transthoracic Echocardiography
LVEF	Left Ventricular Ejection Fraction
IVS	Interventricular Septum
PWT	Posterior Wall Thickness
RV	Right Ventricle
CT	Computed Tomography
T-LBL	T-cell Lymphoblastic Lymphoma
SaO_2_	Oxygen Saturation
IHC	Immunohistochemistry
PAS	Periodic Acid–Schiff
MPO	Myeloperoxidase
MGG	May–Grünwald–Giemsa
TdT	Terminal Deoxynucleotidyl Transferase
HD-AraC	High-Dose Cytarabine
MUD	Matched Unrelated Donor
ATG	Anti-Thymocyte Globulin
GVHD	Graft-Versus-Host Disease
CBC	Complete Blood Count
WBC	White Blood Cell
MRI	Magnetic Resonance Imaging
AVN	Avascular Necrosis
OS	Overall Survival
PFS	Progression-Free Survival
EHA	European Hematology Association
CMML	Chronic Myelomonocytic Leukemia
EBMT	European Society for Blood and Marrow Transplantation
CML	Chronic Myeloid Leukemia
GvL	Graft-Versus-Leukemia
APL	Acute Promyelocytic Leukemia
DLBCL	Diffuse Large B-Cell Lymphoma
EFS	Event-Free Survival
